# BOLD-MRI early detect femoral head osteonecrosis following steroid-treated patients

**DOI:** 10.1097/MD.0000000000008401

**Published:** 2017-11-03

**Authors:** Jing Li, Jingjing Wang, Jihua Zhao, Bin Yuan, Liming Xing, Fengming Tang, Lei Liu, Mingming Lu, Quan Zhang, Jun Zhao, Peng Gu, Jianhui Li, Zhuoli Zhang, Chong Sun, Yu Zhang, Fei Yuan

**Affiliations:** a Department of MRI, Pingjin Hospital, Tianjin; b Department of ICU, Pingjin Hospital, Tianjin; c Department of Medical and Education, Pingjin Hospital, Tianjin; d Department of Orthopedics, Pingjin Hospital, Tianjin; e Department of Radiology, Feinberg School of Medicine, Northwestern University, Chicago, IL; f Department of Orthopedics, Qilu Hospital, Shandong University, Jinan, Shandong; g Philips Healthcare, Beijing, China.

**Keywords:** blood oxygen level-dependent magnetic resonance imaging, femur, glucocorticoids, osteonecrosis

## Abstract

The purpose of the study is to evaluate the feasibility of blood oxygenation level-dependent MRI (BOLD-MRI) to early detect the femoral head osteonecrosis (FHON). One hundred twelve patients were recruited who had received steroid treatment. The normal control group included 10 volunteers with 20 hips. MRI examinations were performed in all patients following up at 1, 4 to 5, 7 to 8, and 12 to 13 months after steroid therapy. With the section cross as the biggest lesion in coronal images, we set 6 regions of interest (ROIs) per section to analyze the morphological performance of routine MRI sequences and the differences of R2∗ values and their dynamic changes of BOLD-MRI between the control and the FHON group. A total of 15 hip joints were diagnosed with FHON. Seven right hips and 8 left hips were affected. In the first and second MRI examinations, the area of the lesion for both conventional MRI and BOLD-MRI R2∗ mapping was difficult to distinguish the lesion border. However, at the third and the fourth MRI examinations, some of the affected regions for R2∗ mapping were larger than those in conventional sequences for the same patient. BOLD-MRI has some significant advantages in early detecting FHON over conventional MRI techniques and it can be feasible noninvasive tool for detecting and evaluating FHON after steroid therapy.

## 1 Introduction

Osteonecrosis is a disease caused by reduced blood flow to bones in the joints.^[1,2]^ Femoral head osteonecrosis (FHON) usually affects people younger than 50 years old and leads to collapse and osteoarthritis, which can reduce the patients’ quality of life.^[3]^ The collapse of femoral head is most damaging to patients with FHON. The hip joint deteriorates and undergoes degenerative changes without effective treatments.^[4]^ Early treatment of FHON may prevent the progression of the disease.^[5]^ Thus, early accurate diagnosis is crucial to improve the prognosis of patients with FHON. The conventional diagnosis modalities include radiography, computed tomography (CT), magnetic resonance imaging (MRI), and radionuclide scanning. Radiography and CT have low sensitivity, and radiative and radionuclide examinations have a high false-positive rate.^[6]^ MRI is sensitive and specific for early diagnosis.^[7]^ FHON has been shown to be correlated with anoxia and ischemia.^[8]^ Functional blood oxygenation level-dependent MRI (BOLD-MRI) can be used to measure vascular oxygenation changes due to neuron activity and kidney blood flow variation because R2∗ mapping is sensitive to oxyhemoglobin change.^[9]^ This study attempted to compare BOLD-MRI with conventional MRI sequences for determining the onset of FHON following steroid-related osteonecrosis and to determine the R2∗ value changes in patients’ lesions of the femoral head when the state of illness progressed.

## 2 Materials and methods

### 2.1 Ethics statement

This retrospective study was approved by the institutional review board of Pingjin Hospital, Tianjin, China. Written informed consent was obtained before collection.

### 2.2 Patient

Our study was performed between March 2012 and March 2014. We recruited 112 patients who had received steroid treatment. All patients were male, with an age range of 17 to 46 years. All the steroid doses were converted to a prednisolone-equivalent dose. The total dose ranged from 666.75 to 103,260 mg, and the length of treatment was 4 to 32 days. MRI examinations were performed for all patients following up at 1, 4 to 5, 7 to 8, and 12 to 13 months after steroid therapy.

Inclusion criteria: patients who were diagnosed with FHON using conventional MRI; patients with a history of intravenous injection of steroids; Association Research Circulation Osseous (ARCO) stage 0, I, or II; no use of any drug in the 2 hours prior to the scan; and no hip pain.

Exclusion criteria: patients were diagnosed without FHON via conventional MR images; ARCO stage III or IV; suffering from multisystem sickness, which can lead to bone ischemia; trauma of the hip; alcoholism; claustrophobia; having any disease that could affect hemodynamics; use of any drug or substance that could affect hemodynamics; and anemia.

Of these patients, 9 showed grossly abnormal findings in single or both femoral heads of early osteonecrosis (ARCO stage I or II). The remaining 103 patients had normal MRI findings or had progressed to ARCO stage III or IV during follow-up. Ultimately, 9 patients were enrolled in the study. The mean age was 21 ± 3 years (range 18–27 years). The total dose of prednisolone-equivalent steroid was 47,173.3 ± 35,500.9 mg (range 12,800–103,260 mg). At the first follow-up visit, 6 hips were normal, and 12 hips were at stage I. A total of 15 hip joints were diagnosed with FHON, and 3 were normal. Seven right hips and 8 left hips were affected. Six patients had bilateral involved and 3 patients had unilateral involved (Table 1).

**Table 1:**
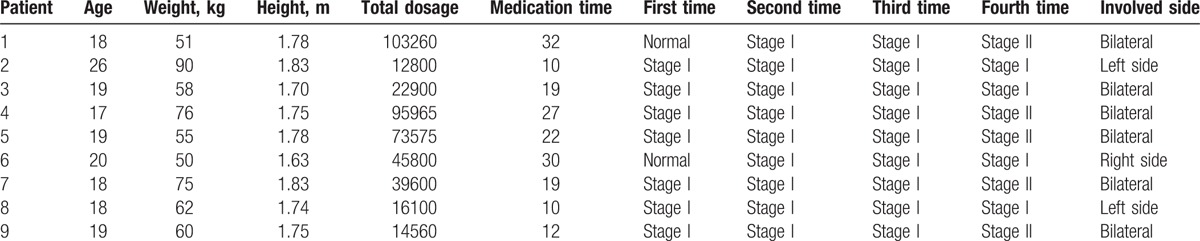
Distribution of clinical and conventional MRI findings of the patient group.

The normal control group included 10 volunteers with 20 hips. All the volunteers did not drink alcohol for the 2 weeks prior to the examinations. Inclusion criteria: male; age between 20 and 30 years; normal hip on conventional MRI; no history of steroid use; without multisystem sickness, which can lead to bone ischemia; no hip trauma history; and no history of alcoholism.

### 2.3 MRI

MRI was performed with a 3.0T system (Achieva Intera; Philips Medical Systems, Best, The Netherlands) using an 8-channel flexible matrix coil to receive the MR signal. The following sequences were applied: coronal T2WI/TSE sequence (TR/TE: 8942/120 ms; field of view [FOV]: 261 × 400 mm; matrix: 280 × 336; 24 slices; FA: 90°; slice thickness: 3 mm; slice gap: 0.3 mm; scan time: 2′41′′); coronal PDW/SPAIR sequence (TR/TE: 4439/30 ms; FOV: 261 × 400 mm; matrix: 476 × 610; 24 slices; FA: 90°; slice thickness: 3 mm; slice gap: 0.3 mm; scan time: 2′41′′); axial T2WI/SPAIR sequence (TR/TE: 8942/120 ms; FOV: 261 × 400; matrix: 280 × 336; 24 slices; FA: 90°; slice thickness: 3 mm; slice gap: 0.3 mm; scan time: 2′41′′); and coronal BOLD T2∗-weighted using a multiecho fast field echo (FFE) sequence (TR: 15 ms; TE: 9.21/18.42/27.63/36.84/46.05/55.26/64.47/73.68 ms; echo: 8; FOV: 250 × 429 mm; matrix: 312 × 429; 8 slices; FA: 30°; slice thickness: 2.5 mm).

### 2.4 Image analysis and creation of R2∗ mapping

**Figure 1:**
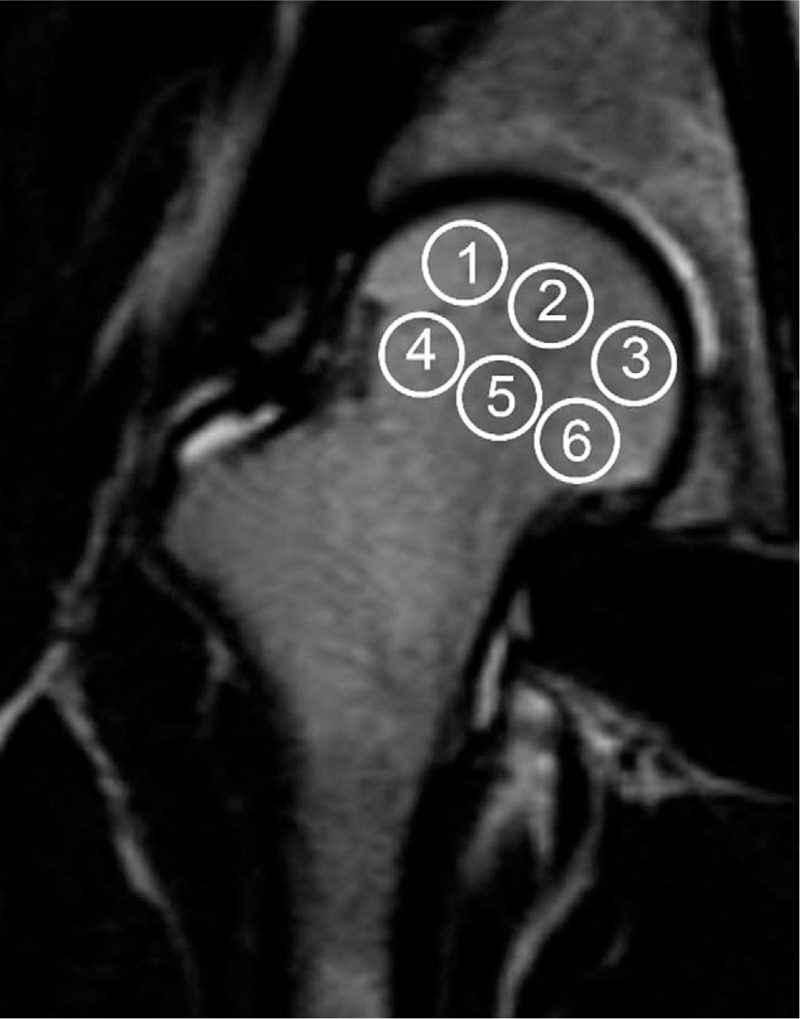
Defining the ROI in the femoral head. The ROI in the femoral head is defined in the FHON group as the section cross with the biggest lesion, and we set 6 ROIs in the section. In the control group, the section crossed the fovea of the femoral head. The ROIs were separated into a 2-tier network and arranged ectoentad. FHON = femoral head osteonecrosis, ROI = region of interest.

Two radiologists with more than 15 years of experience in musculoskeletal radiology evaluated the image data and reached a consensus. The “double-line” sign on the T2WI sequence or the “serpentine-like” geographic border on the SPAIR sequence was considered a pathognomonic sign of FHON. We analyzed BOLD-MRI images based on previously published work.^[10]^ In the FHON group, we set 6 regions of interest (ROIs) in the section crossing the biggest lesion. In the control group, the section crossed the fovea of the femoral head (Fig. 1). Each ROI excluded the femoral head cartilage, the epiphyseal line and the bone cortex. We analyzed morphological performance in routine MR images between the control group and the FHON group. The image stacks acquired at all echo times were loaded into ImageJ software (NIH, Bethesda, MD). We set the ROI as large as possible without exceeding the lesion border. All the above measurements were generated by the same operator, and the average value of 3 measurements was calculated.

After the onset of ischemia, osteocytes begin to disappear within 2 to 5 days and completely disappear within 2 to 4 weeks.^[8]^ Thus, we planned the examination schedule based on MRI examinations at 1, 4 to 5, 7 to 8, and 12 to 13 months after steroid therapy.

At the first examination, 2 patients were normal. Therefore, we divided the first-time detection group into 2 subgroups (the first-time unaffected group and the first-time affected group). Two patients with 3 involved hips were in the first-time unaffected group. The rest were in the first-time affected group. The osteonecrosis lesions were classified and staged according to the ARCO classification criteria by consensus between a musculoskeletal radiologist and an orthopedic surgeon with extensive experience in the diagnosis and treatment of FHON. The R2∗ image performance in each district of the femoral head in the normal group and the differences in the R2∗ values between the normal group and each examination of the patient group were analyzed. The R2∗ value change in a patient at different times and the difference between the R2∗ image and routine sequences in the lesion area of 1 patient were also explored.

### 2.5 Statistical analysis

In this study, all statistical analyses were performed using GraphPad Prism version 6 software (GraphPad Prism Software Inc, San Diego, CA). A *P* value <.05 was considered statistically significant. Before statistical analysis, a normal R2∗ distribution was obtained graphically and was statistically analyzed using the Shapiro-Wilks test. The results indicated that the data for zone 2, 5, and 6 were not normally distributed. The data for zone 2 and 6 fit the normal distribution curve after logarithmic transformation. The nonparametric Brown-Forsythe test was used to analyze the zone 5 data. No statistically significant differences were found in the Brown-Forsythe test, indicating that there was approximately equal variance for this dependent variable with the other zones. One-way analysis of variance (ANOVA) was applied to identify any significant differences between the mean R2∗ values of the osteonecrosis lesions in the other zones.

## 3 Results

### 3.1 Conventional MRI images of control and FHON group

Conventional MRI showed no collapse of the femoral head and no dislocation and subluxation of the femoral head in either the control group or the FHON group. None of the enrolled people showed injury to the ligaments, femoral neck hernia concave, or femoroacetabular impingement. At the first examination, 2 patients with 3 involved hips were normal and 7 patients with 12 involved hips were diagnosed with FHON. At the second and third examinations, all patients with 15 involved hips were affected by FHON, and all of them were classified as ARCO stage I. At the fourth examination, 4 patients with 5 involved hips were classified as ARCO stage I, and 5 patients with 10 involved hips were classified as ARCO stage II. Among all zones, zone 1, 2, and 4 were the most common lesion affected by FHON.

### 3.2 BOLD-MRI mapping of control and FHON group

**Figure 2:**
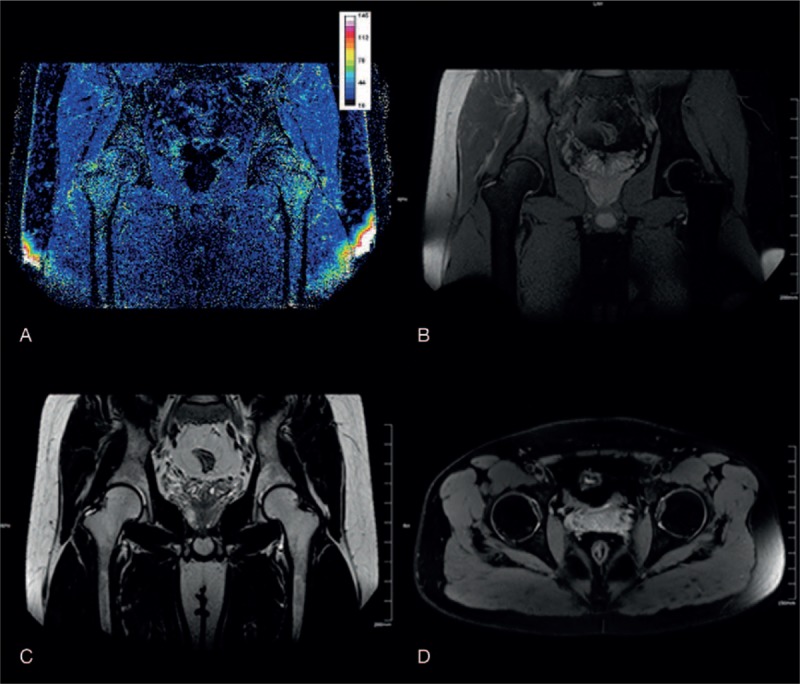
The BOLD-MRI mapping and conventional MR image of a healthy volunteer. (A) R2∗ mapping reflected the structure and blood supply variation of the femoral head. The values of zones 4 and 1 were highest, followed by zones 6 and 3, and zones 5 and 2 had the lowest values. Conventional MRI (B–D, coronal PDW/SPAIR, coronal T2WI/TSE, axial T2WI/SPAIR) showed a normal femoral head without any disease. BOLD-MRI = blood oxygen level-dependent MRI, R2∗ = total transverse relaxation time, TSE = turbo spin echo.

BOLD-MRI mapping of the control group: R2∗ mapping reflected the structure and the blood supply variation of the femoral head. The values of zone 4 and 1 were the highest, followed by zone 6 and 3, and zone 5 and 2 had the lowest value (Fig. 2). In the control group, the R2∗ values of zone 1 to 6 were 22.54 ± 2.28, 16.20 ± 1.77, 19.21 ± 1.70, 24.74 ± 2.27, 18.31 ± 3.45, and 21.37 ± 2.44 kHz, respectively (Table 2).

**Table 2:**
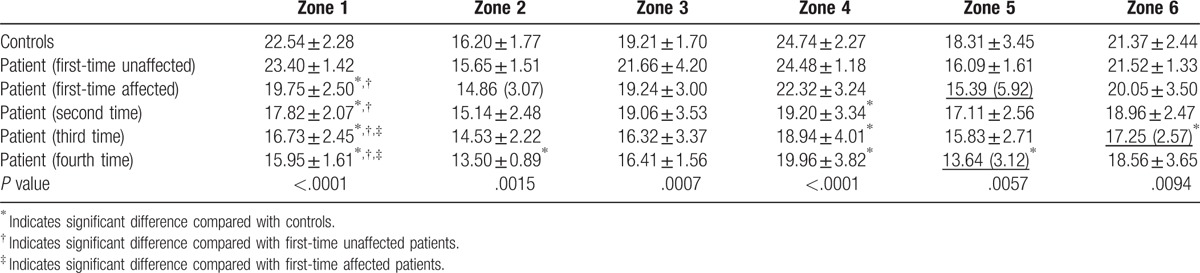
R2∗ values (without underline: mean ± SD; underline: median and interquartile range) in zones 1 to 6 for the control and patient groups.

**Figure 3:**
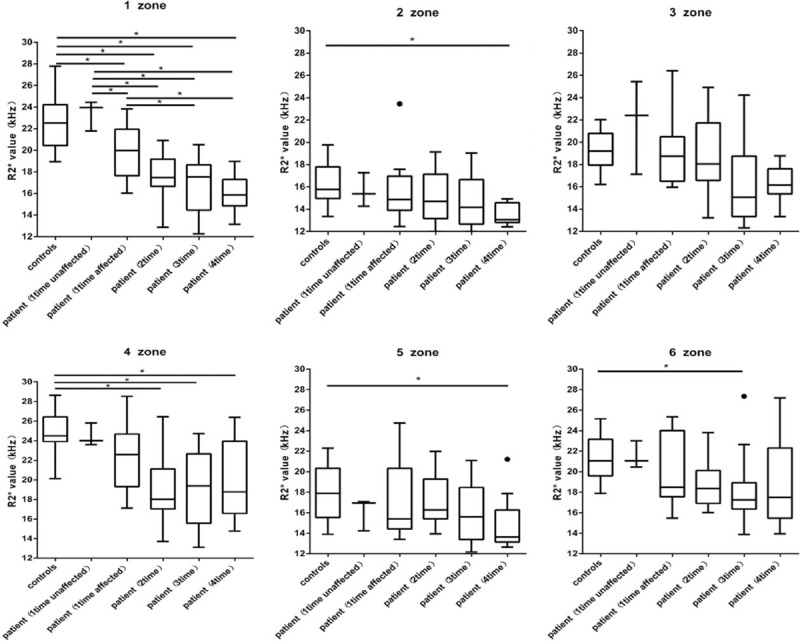
Box plot diagram of R2∗ values in all zones, revealing a marked decline in R2∗ when the control group and the FHON group were compared 4 times. In zone 1, there was a significant decrease in the R2∗ value at the first comparison and a continuous decrease at the other time points. FHON = femoral head osteonecrosis, R2∗ = total transverse relaxation time.

BOLD-MRI mapping of the FHON group: The R2∗ value in the FHON group decreased (Fig. 3). The lesion had a lower signal than the normal area in the FHON group. According to conventional MRI, zone 1 and 4 were the predominantly involved zones. In the affected femoral head, R2∗ value decreased significantly in zones 1 and 4. Moreover, zone 1 was the earliest zone involved on BOLD-MRI. The R2∗ value was significantly lower in the patient group than in the control group after steroid treated 1 month later (patient vs control: 19.75 ± 2.50 vs 22.54 ± 2.28, *P* < .0001), and the R2∗ decreased gradually during the follow-up period. Zone 3 did not show any significant differences between the control and patient groups. There were significant differences during the last examination between the patient group and control group in all zones except zone 3 and 6 (Table 2).

### 3.3 Differences in the lesion area between R2∗ mapping and conventional MR images

**Figure 4:**
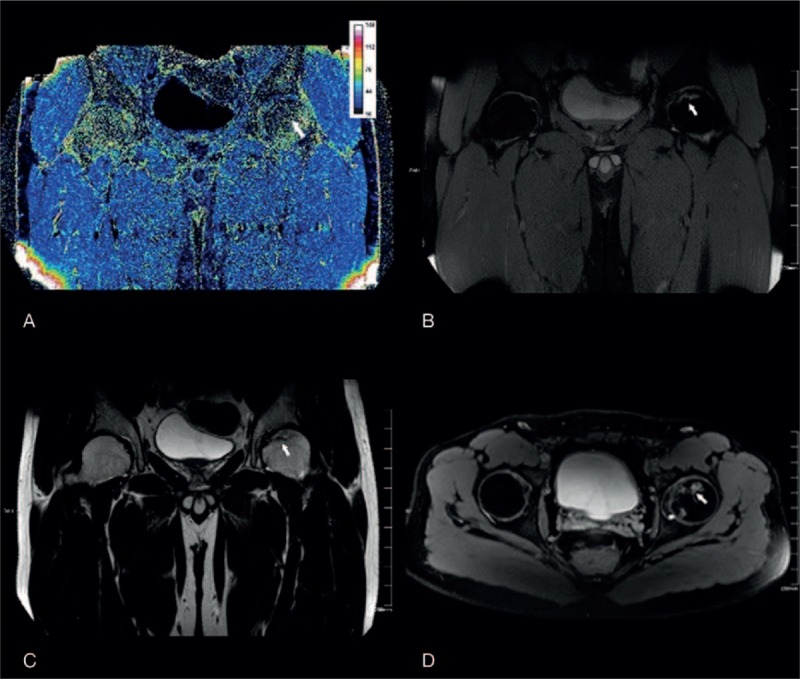
BOLD-MRI mapping and conventional MR image of a unilateral FHON patient. (A) R2∗ mapping reflected a lesion (white arrow) with the decreased value in the left side. The values of zones 1 to 3 declined. Conventional MRI (B–D, coronal PDW/SPAIR, coronal T2WI/TSE, axial T2WI/SPAIR) showed the lesion with a “double-line” sign on the T2WI sequence and a “serpentine-like” geographic border on the SPAIR sequence. The region of the lesion (white arrow) was obviously smaller than indicated by R2∗ mapping. BOLD-MRI = blood oxygen level-dependent MRI, FHON = femoral head osteonecrosis, R2∗ = total transverse relaxation time.

In the first and second scans, the area of the lesion for both conventional MRI and R2∗ mapping was approximate. It was difficult to distinguish the lesion border, especially for R2∗ mapping. However, at the third and the fourth examinations, some of the affected regions for R2∗ mapping were larger than those in conventional sequences for the same patient (Fig. 4).

### 3.4 Dynamic changes in the R2∗ value

**Figure 5:**
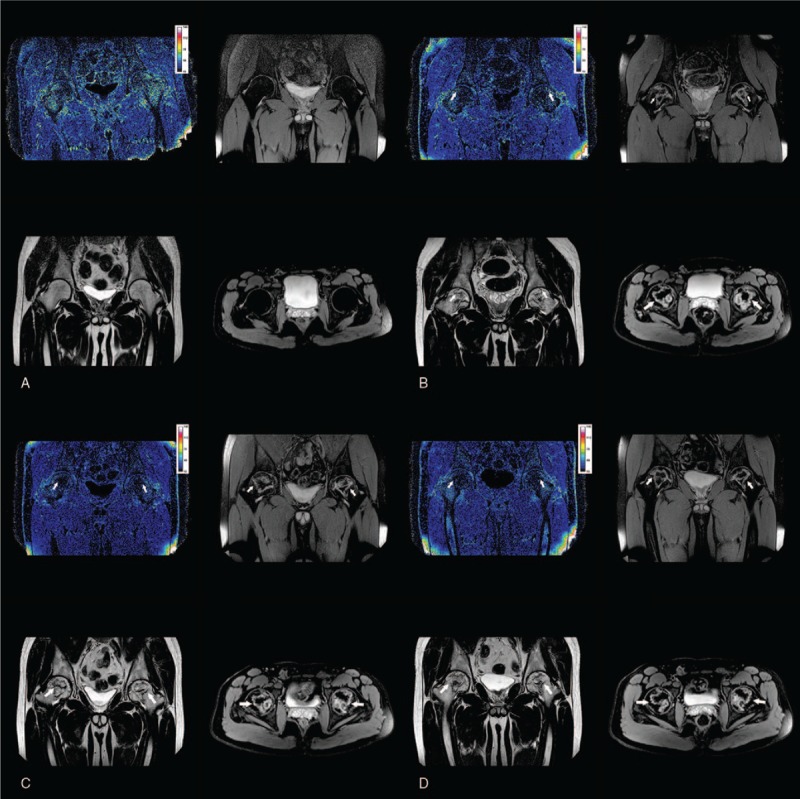
Dynamic changes in the R2∗ value and conventional MRI representation of femoral head over 4 examinations of the same patient. (A) The MRI findings at 1 month after steroid treated, which showed a slight increase of R2∗ value of the femoral head. However, the conventional MRI (coronal PDW/SPAIR, coronal T2WI/TSE, and axial T2WI/SPAIR) appeared normal. (B) The MRI findings at 4 months after steroid treated, which showed that a drop in R2∗ value of the femoral head. The lesion with a “double-line” sign on the T2WI sequence, the “serpentine-like” geographic border on the SPAIR sequence with conventional MRI. (C) The MRI findings at 7 months after steroid treated, which showed that a further decrease in R2∗ value of the femoral head. And the increase of “serpentine-like” geographic border on the SPAIR sequence with conventional MRI. (D) The MRI findings at 12 months after steroid treated, which showed that the R2∗ value of the femoral head decreased again to the minimum. And on the SPAIR sequence the “serpentine-like” geographic border grew up to the maximum size. R2∗ = total transverse relaxation time

In the FHON group, there were 2 patients (3 involved hip joints) who did not show any abnormal changes in the conventional MRI at the first examination (after 1 month of corticosteroid usage). When we used R2∗ mapping to examine the 2 patients, the R2∗ value slightly increased in zone 1 at the first detection. However, when the R2∗ value decreased, the lesions were apparent on conventional MRI. Over time, the R2∗ of all the zones showed a decreasing trend. Compared with the control group, the R2∗ value of all patient groups showed significantly decreased in zone 1, except for the patient whose femoral head was unaffected on conventional MRI. All zones demonstrated significant decrease at the third or fourth examinations except zone 3 (Fig. 5).

## 4 Discussion

The femoral head is supplied by 3 main groups of arteries, including the posterior superior nutrient arteries of the femoral head arising from the deep branch of the medial femoral circumflex artery (MFCA), a posterior inferior nutrient artery from the main branch of the MFCA, and the piriformis branch of the inferior gluteal artery. The path mechanism of corticosteroid-associated FHON remains unclear. There are many potential explanations, such as genetic predisposition, fat metabolic disorders, hypofibrinolysis and thrombophilia,^[11]^ impaired angiogenesis,^[12]^ osteocyte death,^[8]^ and intraosseous hypertension.^[13]^ The pathophysiology of corticosteroid-associated FHON is complicated. Corticosteroids can cause bone marrow stem cells transforming into fat cells and promote their hypertrophy, which could increase the intraosseous pressure. Intraosseous hypertension could give rise to venous sinusoidal compression and microvascular coagulation in the proximal femur. In addition, corticosteroids could hinder angiogenesis by reducing the levels of vascular endothelial growth factor.

When FHON occurs, it is usually located at the peripheral zone of the lateral epiphyseal artery, where the subchondral bone in the anterolateral quadrant of the femoral head is usually wedge-shaped.^[14]^ Using super-selective angiography, Atsumi et al^[15]^ indicated that the blood supply of the superior retinacular arteries from the extra-osseous site was injured when FHON occurred. In our research, zone 1 and 4 corresponded to the locations at which the lateral epiphyseal artery supplies the blood flow.

In patients with a subcapital fracture, the vascularity of the femoral head only depends on perfusion from the ligamentum teres, which normally provides the blood supply to 10% to 20% of the femoral head.^[6]^ In our research, the R2∗ values of zone 2 and 5 were the lowest in the control group, indicating that these zones usually have a greater blood supply than the other zones, with or without FHON.

In our study, the R2∗ value in the FHON group decreased compared with the control group for the following reasons. First, the decreases in blood flow and blood volume were due to vessel occlusion of the femoral head, which caused a decrease in the content of both deoxyhemoglobin and oxyhemoglobin. Second, the decreasing ratio of deoxyhemoglobin to oxyhemoglobin was due to the reduction in oxygen consumption, which caused cellular apoptosis in the necrotic area. Our study indicated that the mechanism is the same as the kidney with total arterial occlusion and evident renal atrophy.^[16]^

In BOLD-MRI studies of the kidney, an increase in R2∗ prompts an increase in the ratio of deoxyhemoglobin to oxyhemoglobin. However, an increase in the R2∗ value implies that deoxyhemoglobin might increase or oxyhemoglobin might decrease. In patients with high grade renal artery stenosis but with preserved kidney volume whose kidney has a normal shape, the rising R2∗ value suggests that renal oxygenation decreases and deoxyhemoglobin increases.^[16]^ In our study, there were 2 cases with definite lesions using conventional sequences in the fourth month after corticosteroid treatment. Compared with the control group, the 2 cases did not show obviously abnormal findings in conventional MRI. However, the R2∗ value increased when they underwent the first examination after corticosteroid treatment. Similar to the patients with severe renal artery stenosis, we speculated that the blood supply decreased when the blood vessels were partially blocked, while the bone tissue allowed sufficient oxygen intake from the blood, which caused an increase in deoxyhemoglobin and a decrease in oxyhemoglobin.

In the ARCO, Ficat and University of Pennsylvania classification systems, stage 0 means that all diagnostic techniques are normal or nondiagnostic except histology. Hungerford et al^[17]^ termed this stage “silent hip” because there have been no appropriate techniques for diagnosis. In our research, 2 patients with 3 involved hips manifested as normal in conventional sequences. We speculated that the 2 cases can classify as stage 0. At that time, their R2∗ values showed a slight increase compared with the control group and a significant difference compared to the other patient groups. This tendency indicates that R2∗ mapping has good prospects for the early diagnosis and detection of osteonecrosis of the femoral head. However, we need more samples to further verify our findings and to confirm the cutoff value.

In our study, all patients demonstrated necrosis of the femoral head in the first month after corticosteroid treatment, except for the 2 cases that were previously mentioned. The R2∗ value was lower than that of the control group at the same time. During follow-up, we could detect a “double-line sign,” which was a characteristic of FHON in the conventional MRI sequences.

The R2∗ value continually declined from the second to the fourth detection. There might be 2 reasons for this phenomenon. First, in necrosis, the blood supply reduced to the lowest limitation, which caused deoxyhemoglobin and oxyhemoglobin decrease. Second, outside the necrosis area, the ratio of deoxyhemoglobin to oxyhemoglobin further decreased during the appearance of granulation tissue angiogenesis. The area of R2∗ value decreasing is larger in patients whose necrosis border did not demonstrate angiogenesis of granulation tissues. The infarct was well delimited from the surrounding bone by a thin hyperemic border.^[8]^ Most existing studies have suggested that once the sclerosis band occurs, the femoral head necrotic lesion cannot be repaired with granulation tissue, although the sclerosis band could provide biological support and increase tissue hardness. If we could detect the lesion earlier, we could implement effective intervention measures, and improve outcomes. In our study, R2∗ mapping slightly increased, when the conventional MRI did not show any abnormal changes. Thus, R2∗ mapping would be a potential measure on early diagnosis of FHON.

To avoid the disorder of the magnetic field in the ischemic area that caused the R2∗ decrease around the necrosis zone, we examined one patient with both R2 mapping and R2∗ mapping. Because R2 mapping is not influenced by disorder of the magnetic field, we could rule out this phenomenon.

One limitation of our study was the lack of histology data corresponding to MRI data so we have no direct information about the biological basis of the MRI change. However, this does not impact the diagnostic value of our results. Second, BOLD-MRI has some advantages. But PET-MR will be introduced in clinical scenarios. Even more, functional BOLD-MRI will merge with PET tracers to further increase molecular imaging as a relevant medical discipline. Multimodality imaging techniques will play a leading role in relevant clinical applications. Finally, all the cases were young people, thus there is lack of other age groups. However, our research provides the first characteristic of glucocorticoids related early osteonecrosis of the femoral head with BOLD-MRI. We use conventional MRI and BOLD-MRI with 4 times follow-up, which revealed the dynamic progress of early osteonecrosis of the femoral head.

To the best of our knowledge, this is the first study to use BOLD-MRI to assess FHON. The study showed that BOLD-MRI can quantify FHON. And this technology has clinical potential for detecting necrosis of the femoral head in the early stage. This study compared the femoral head of FHON with the healthy control. Self-control group can be used as an alternative to evaluate the FHON before and after steroid use in future, on account of the variation of age, and the difference between individuals.
